# Ultrafast and accurate sequence alignment and clustering of viral genomes

**DOI:** 10.1038/s41592-025-02701-7

**Published:** 2025-05-15

**Authors:** Andrzej Zielezinski, Adam Gudyś, Jakub Barylski, Krzysztof Siminski, Piotr Rozwalak, Bas E. Dutilh, Sebastian Deorowicz

**Affiliations:** 1 Department of Computational Biology, Faculty of Biology, Adam Mickiewicz University, Poznan, Poland; 2 Faculty of Automatic Control, Electronics and Computer Science, Silesian University of Technology, Gliwice, Poland; 3 Department of Molecular Virology, Faculty of Biology, Adam Mickiewicz University, Poznan, Poland; 4 Institute of Biodiversity, Faculty of Biological Sciences, Cluster of Excellence Balance of the Microverse, Friedrich Schiller University Jena, Jena, Germany; 5 Theoretical Biology and Bioinformatics, Science4Life, Utrecht University, Utrecht, the Netherlands

**Keywords:** Software, Metagenomics, Genome informatics, Classification and taxonomy, Comparative genomics

## Abstract

Viromics produces millions of viral genomes and fragments annually, overwhelming traditional sequence comparison methods. Here we introduce Vclust, an approach that determines average nucleotide identity by Lempel–Ziv parsing and clusters viral genomes with thresholds endorsed by authoritative viral genomics and taxonomy consortia. Vclust demonstrates superior accuracy and efficiency compared to existing tools, clustering millions of genomes in a few hours on a mid-range workstation.

## Abstract (web summary)

Vclust generates fast and accurate estimation of average nucleotide identity for viral genomes, scaling clustering to millions of genomes.

## Main

Metagenomics and viromics are identifying new viruses at an unprecedented rate, but recognizing which sequences were seen before remains challenging^1,2^. Calculating average nucleotide identity (ANI), essential for classification, is limited by the scalability of alignment tools like anicalc^3^, commonly used to cluster viruses into virus operational taxonomic units (vOTUs), or VIRIDIC^4^, recommended by the International Committee on Taxonomy of Viruses (ICTV) to delineate bacteriophage species and genera. Large-scale sequence comparisons rely on efficient, albeit less accurate, *k*-mer approaches such as sketching (FastANI^5^) or sparse approximate alignments (skani^6^). Moreover, most tools lack clustering functionality or do not scale to large metagenomic datasets (Extended Data Table 1).

**Fig. 1:**
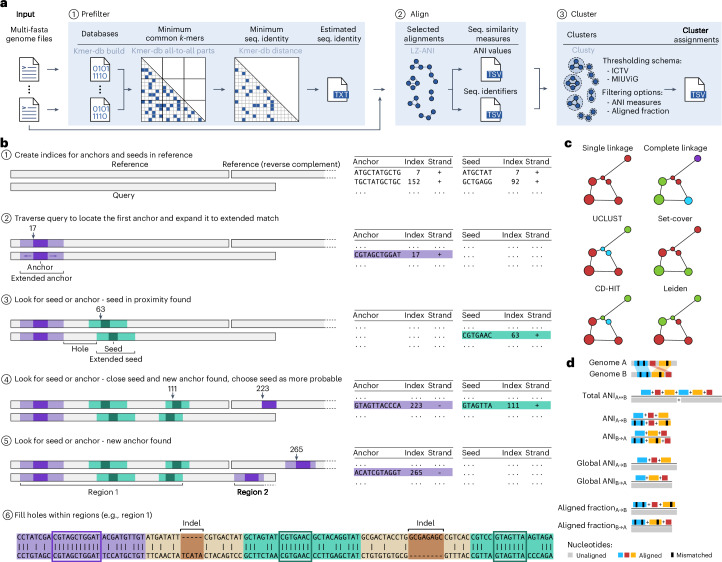
Vclust algorithm and features. **a**, Vclust’s workflow: (1) prefilter similar genome sequence pairs with sufficient *k*-mer-based identity estimated using Kmer-db 2; (2) align similar genome pairs and calculate ANI using LZ-ANI; and (3) cluster genomes based on defined cutoffs using Clusty. **b**, Sequence alignment using Lempel–Ziv parsing (Methods). **c**, Vclust’s clustering algorithms. Vertex size represents genome sequence length, and edge lengths indicate the distance (1 − ANI) between genomes. A more detailed depiction of the clustering algorithms is shown in Extended Data Fig. 1. **d**, Illustration of the calculation of Vclust’s sequence similarity measures.

Vclust is a fast alignment-based method that calculates ANI measures for complete and fragmented viral genomes and clusters them according to ICTV and Minimum Information about an Uncultivated Virus Genome (MIUViG) standards^1,4^ (Extended Data Table 1). It introduces three components (Fig. 1a). First, Kmer-db 2, a successor of Kmer-db^7^, rapidly determines related genomes using either all *k*-mers or a predefined fraction. Second, LZ-ANI, a Lempel–Ziv parsing-based algorithm (Fig. 1b and Methods), identifies local alignments within related genome pairs and calculates overall ANI from these aligned regions with high sensitivity and accuracy. Third, Clusty efficiently implements six clustering algorithms suited for sparse distance matrices with millions of genomes (Fig. 1c).

**Fig. 2:**
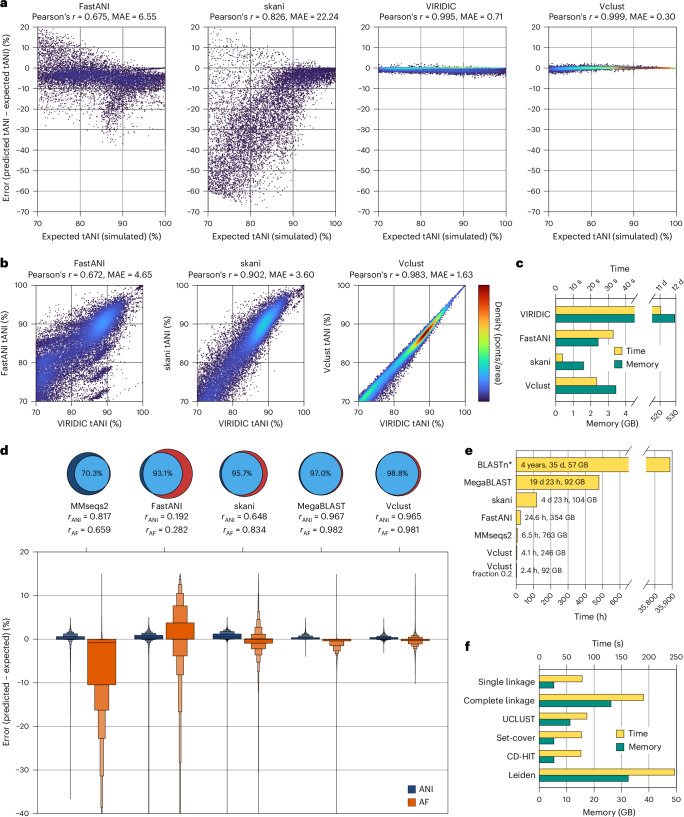
Comparison of Vclust with other tools on various datasets. **a**, Difference between predicted and expected tANI values for 10,000 bacteriophage genome pairs with simulated mutation events. **b**, Correlations with VIRIDIC tANI values for 22,607 complete bacteriophage genome pairs. **c**, Wall time and peak memory usage for processing 4,244 bacteriophage genomes (32 threads). Vclust and VIRIDIC include clustering, while FastANI and skani only calculate ANI. **d**, Venn diagrams comparing numbers of contig pairs meeting MIUViG thresholds (ANI ≥ 95% and AF ≥ 85%) predicted by BLASTn (purple) and other tools (red). The boxen plot shows the error distribution of predicted ANI and AF values relative to corresponding BLASTn-based reference values for 4,361,743 contig pairs meeting MiUVUG thresholds. The center line denotes the median, while each box level from the median contains half of the remaining observations. **e**, Wall time and peak memory usage for calculating ANI and AF among 15,677,623 IMG/VR contigs (64 threads). BLASTn values were estimated from a random sample of 1,000 query contigs. Vclust was tested in its default setting and with a 0.2 fraction of *k*-mers used at the ‘prefilter’ step. **f**, Wall time and peak memory usage of Vclust’s clustering algorithms for grouping IMG/VR contigs into vOTUs. Source data

We first tested Vclust’s accuracy of total average nucleotide identity (tANI) estimation (Fig. 1d) among 10,000 pairs of phage genomes containing simulated mutations, including substitutions, deletions, insertions, inversions, duplications and translocations (Methods and Supplementary Table 1). Vclust and VIRIDIC, both alignment-based tools, provided tANI values close to the expected ones, with mean absolute error (MAE) values of 0.3% and 0.7%, respectively, outperforming FastANI (6.8%) and skani (21.2%; Fig. 2a). Vclust predictions consistently approached expected values as tANI increased, while VIRIDIC underestimated tANI (Fig. 2a). Among genome pairs above the ICTV’s species threshold (tANI ≥ 95%^4^, *n* = 1,188), Vclust reported only 22 pairs below the threshold, whereas VIRIDIC underestimated nearly 10× more (*n* = 210; Supplementary Table 2).

Next, we determined tANI using VIRIDIC in an all-to-all comparison of 4,244 bacteriophage genomes. Vclust had a higher correlation with VIRIDIC tANI (Pearson’s *r* = 0.983) than skani (*r* = 0.902) and FastANI (*r* = 0.671) across the entire tANI range ≥ 70% (22,606 genome pairs; Fig. 2b) and outperformed both tools within their reliability range ≥ 80%^5,6^.

Then, we compared the consistency of the bacteriophage species groupings (tANI ≥ 95%) with the official ICTV taxonomy (Methods). Vclust and VIRIDIC showed moderate agreement with ICTV (73% and 69%, respectively), followed by FastANI (40%) and skani (27%). Upon examining genome pairs where both Vclust and VIRIDIC diverged from the ICTV’s classification, we found inconsistencies in 50 ICTV taxonomic proposals (Supplementary Tables 3 and 4). Excluding these cases improved the agreement of both tools with ICTV taxonomy, with Vclust retaining superiority (95%) over VIRIDIC (90%) and the other tools (Supplementary Table 5). For genus groupings (tANI ≥ 70%), Vclust achieves 92% agreement with ICTV taxonomy, comparable to VIRIDIC’s 93%, despite inconsistent application of the threshold we found across ICTV genera (Supplementary Tables 6 and 7 and Extended Data Fig. 2). Given Vclust’s high agreement with ICTV taxonomy, accurate tANI determination and processing speed >40,000× faster than VIRIDIC (Fig. 2c and Supplementary Table 8), it emerges as the prime tool for bacteriophage classification.

We then assessed Vclust’s accuracy in matching contig pairs that satisfy MIUViG thresholds (ANI ≥ 95% and aligned fraction (AF) ≥ 85%; Fig. 1d). We subsampled over 90,000 metagenomic contigs from the IMG/VR database and used BLASTn^8^+ anicalc^3^ (most accurate alignment-based method) to identify over 4 million sequence pairs that met MIUViG thresholds. Vclust recovered the highest number of pairs (99%), followed by MegaBLAST + anicalc (97%), skani (96%, or 86% in the fastest mode), FastANI (96%) and MMseqs2 (ref. ^9^) (70%; Fig. 2d and Supplementary Table 9). Both Vclust and MegaBLAST produced ANI and AF estimates consistently with the BLASTn values (Pearson *r* > 0.96), outperforming the other tools (*r* = 0.2–0.8). On average, ANI and AF values obtained by Vclust and MegaBLAST showed minimal deviation from the expected values (MAE < 1%; Supplementary Table 9), with Vclust having the narrowest error range among all the tools (Fig. 2d). This trend is consistent across varying contig sizes, from smallest (<5 kb) to largest (>100 kb; Supplementary Table 10).

The scalability of the tools was tested using the entire IMG/VR database of 15,677,623 virus contigs. Vclust performed sequence identity estimations for ~123 trillion contig pairs and alignments for ~800 million pairs, resulting in 5–8 million vOTUs depending on the clustering algorithm (Supplementary Table 11 and Supplementary Fig. 1). These vOTUs are generally consistent with those identified by MegaBLAST, with Vclust clustering approximately 75,000 more contigs on average, indicating higher sensitivity (Supplementary Table 11). Vclust was >115× faster than MegaBLAST, >6× faster than skani or FastANI, and ~1.5× faster than MMseqs2 (Fig. 2e,f, Extended Data Fig. 3 and Supplementary Table 12). Although skani in its fastest mode was 7× faster than Vclust (Supplementary Table 12), it was substantially less accurate (Supplementary Table 9). In addition, Vclust’s runtime and memory usage can be further reduced by ~40% and ~60%, respectively, by analyzing 20% of the *k*-mers in each genome during prefiltering (Fig. 2e), with negligible impact on sensitivity and specificity (Extended Data Fig. 4).

In conclusion, Vclust surpasses the current state-of-the-art methods in viral genome comparison in both accuracy and speed, remaining effective in datasets of millions of sequences. It provides a complete solution for calculating intergenomic similarities and clustering complete, partial and circularly permuted (Extended Data Fig. 5) virus genomes using various ANI measures and clustering algorithms. Given the astonishing diversity of viruses in metagenomic data, we believe that Vclust will be essential for large-scale dereplication and taxonomic classification of viral sequences. It is freely available on GitHub, with a web service option for smaller projects (https://www.vclust.org/), and its core components—Kmer-db, LZ-ANI and Clusty—are available as stand-alone tools for broader applications in sequence comparison and general clustering tasks. Similar to other tools^6^, Vclust’s performance may decrease with large datasets of highly similar genomes owing to the high number of sequence pairs requiring alignment and clustering after prefiltering (Methods). Future work will focus on improving scalability for large homogeneous datasets, including bacterial genomes, and implementing amino acid-based computations (for example, average amino acid identity).

## Methods

### Overview

Vclust is a workflow that introduces and integrates three tools:

1. Kmer-db 2: performs the initial *k*-mer-based estimation of sequence identity of all genome pairs (‘Sequence identity estimation: Kmer-db 2’).
2. LZ-ANI: aligns sequence pairs with nucleotide identity exceeding a specified threshold and calculates ANI and AF measures (‘Sequence alignment: LZ-ANI’ and ‘Calculating ANI and AF’).
3. Clusty: clusters sequences based on ANI and/or AF criteria (‘Clustering sequences: Clusty’).

We implemented Kmer-db 2, LZ-ANI and Clusty in C++20 as stand-alone tools, adaptable for various sequence comparison and clustering tasks (‘Code availability’). Vclust, a Python script, integrates these tools to calculate and cluster viral genomic sequences (‘Vclust implementation’).

### Sequence identity estimation: Kmer-db 2

Kmer-db 2 is an updated tool for *k*-mer-based estimation of pairwise similarities among nucleotide sequences, using either all or a selected fraction of *k*-mers. Unlike fixed-sized sketching (used, for example, by Mash^10^), Kmer-db 2 retains a proportional fraction of *k*-mers per genome, preserving the relationship between sequence lengths.

Kmer-db 2 introduces several improvements enabling the processing of tens of millions of sequences. First, unlike its predecessor, which stored similarity values in RAM as a dense matrix^7^, Kmer-db 2 uses sparse matrices that retain only nonzero elements in all-to-all pairwise genome comparison mode (‘all2all-sp’), allowing it to handle large and diverse genome sets. Second, Kmer-db 2 supports genome datasets partitioned into multiple input files, each generating a separate Kmer-db database. A new mode, ‘all2all-parts’, calculates shared *k*-mers within and across databases, optimizing memory by loading one or two databases into RAM sequentially, although at the expense of additional computational time from repeated database loading. Third, Kmer-db 2 further minimizes RAM usage by storing only genome pairs that meet a minimum threshold of shared *k*-mers and sequence identity. Finally, all modes in Kmer-db 2 support multithreading, except for the distance calculation step, which is sufficiently fast without parallelization. Supplementary Fig. 2 shows the computational performance improvements of Kmer-db 2 over Kmer-db 1, with runtime reductions of 3× to 100× across modes and substantially lower RAM requirements.

### Sequence alignment: LZ-ANI

The LZ-ANI algorithm uses Lempel–Ziv parsing^11^ to align two sequences (the query and the reference).

First, the algorithm constructs two indices (dictionaries): for anchors and seeds. The anchor index maps all *a*-mers (substrings of length *a*) from both strands of the reference sequence to their positions, while the seed index performs the same mapping for shorter *s*-mers (Fig. 1b, step 1).

Next, the query is read from left to right using a sliding window of *a* nucleotides, moving one nucleotide at a time. The parsed *a*-mers are used to search the anchor index for matches in the reference. Upon finding an exact match, the algorithm extends it in both directions (Fig. 1b, step 2). In each direction, a window of size *aw* slides until it encounters more than a certain number of mismatches (*am*) at a time. Then, the extensions of terminal windows are trimmed to remove poorly aligned ends until they have at least *ar* exactly matched nucleotides. This extended anchor initiates the first ‘region’, which corresponds to a local alignment, and is constructed as described below.

The algorithm then moves to the next nucleotide after the extended anchor and looks for *a*-mers (anywhere in the reference) and *s*-mers (within *r* nucleotides from the end of the extended match in the reference) in the dictionaries. Four scenarios may arise:

1. No anchor or seed is found: shift by one position in the query and repeat the process of finding a new anchor or a seed match. However, if the distance in the query between the current position and the end of the previous match exceeds *q* nucleotides, the seed search is discontinued.
2. Only a seed match is found: extend the seed similarly to the initial anchor match, append it to the region, and continue the search for a new anchor or seed match (Fig. 1b, step 3).
3. Only an anchor match is found: close the current region and extend the anchor match to initiate a new region (Fig. 1b, step 5).
4. Both anchor and seed matches are found: select the match less likely to occur by chance, based on their lengths, seed proximity (*r* nucleotides) and the reference sequence length, leading to either scenario 2 or 3 (Fig. 1b, step 4).

Upon closing a region, the algorithm realigns the nucleotide stretches between all the extended matches within the region (Fig. 1b, step 6). This realignment aims to maximize the number of matching nucleotides between neighboring extended matches by allowing a single multi-symbol insertion in the reference or query sequence. As a result, the region represents a local alignment containing both matched and mismatched nucleotides, along with approximated indel fragments. To remove spurious alignments, regions shorter than *g* nucleotides are excluded from further analysis.

The LZ-ANI tool reads input sequences and stores them in RAM in a compact format with three nucleotides per byte. The tool processes sequences in parallel, with each thread comparing a reference sequence to all other sequences. By default, the tool performs all-versus-all pairwise alignments, but it can also accept a filter specifying sequence pairs to align, such as a file generated by Kmer-db (used by Vclust by default).

### Alignment parameters

LZ-ANI parameters are adjustable and were optimized for virus genome sequences (Extended Data Table 2). The default anchor length was set to 11 nucleotides, matching the BLASTn default word size, which provides greater sensitivity than MegaBLAST’s 28-nucleotide word size. The remaining LZ-ANI parameters were optimized using Bayesian optimization with Gaussian process minimization. This optimization involved 100 evaluations on a dataset of 10,000 pairs of complete genomes with simulated mutations (that is, substitutions, insertions, deletions, duplications, inversions and translocations) and known expected ANI values of ≥70% (Supplementary Table 13). The default Vclust parameters were selected based on the lowest MAE between the predicted and reference tANI values. Supplementary Fig. 3 compares the length, number and identity of alignments generated by Vclust (using default parameters), BLASTn and MegaBLAST.

### Calculating ANI and AF

Similarly to BLAST-based ANI methods^3,4^, LZ-ANI alignment between query (*A*) and reference (*B*) encompasses ‘regions’, analogous to BLAST’s high-scoring segment pairs. This alignment allows direct calculation of:

- *L*(*A*, *B*)—the total length (sum) of all regions when aligning query *A* to reference *B*, in nucleotides
- *M*(*A*, *B*)—the total number of matching nucleotides in all regions

These values are used to compute seven sequence similarity measures as follows:

1. ANI for *A* and *B*: $\frac{M(A,\,B)}{L(A,\,B)}$
2. ANI for *B* and *A*: $\frac{M(B,\,A)}{L(B,\,A)}$
3. AF of query *A* to reference *B*: $\frac{L(A,\,B)}{{|A|}}$
4. AF of query *B* to reference *A*: $\frac{L(B,\,A)}{{|B|}}$
5. Global ANI for *A* and *B*: $\frac{M(A,\,B)}{{|A|}}$
6. Global ANI for *B* and *A*: $\frac{M(B,\,A)}{{|B|}}$
7. Total ANI: $$\frac{M(A,\,B)+M(B,\,A)}{{|A|}+{|B|}}.$$

### Clustering sequences: Clusty

Clusty is a versatile package facilitating rapid clustering across diverse data types, using six algorithms: single linkage, complete linkage, UCLUST^12^, greedy set cover^9^, CD-HIT^13^ and Leiden^14^. Our implementations of these algorithms were optimized for sparse distance matrices. A linear memory complexity with the number of distances allows the clustering of tens of millions of objects, provided the matrix remains sufficiently sparse.

Clusty uses threshold-based clustering, assigning an object to a cluster if its distance from the cluster does not exceed a user-defined threshold. Depending on the algorithm, this distance can refer to the closest, furthest or centroid member. While UCLUST, greedy set cover and CD-HIT are inherently threshold-based algorithms, single and complete linkage algorithms construct dendrograms that can be pruned at customizable distance thresholds. Clusty’s sparse data representation assumes all input values to meet the distance or similarity threshold. However, the tool allows clustering data at more stringent thresholds through additional filtering of any combinations of distance/similarity values (for example, tANI, ANI and AF) and/or other measure values (for example, minimum/maximum number of alignments, minimum/maximum number of matched nucleotides). Consequently, the matrix provided to Clusty does not need to be sparse; the tool can handle dense matrices and apply filtering at the loading stage.

**Extended Data Fig. 1:**
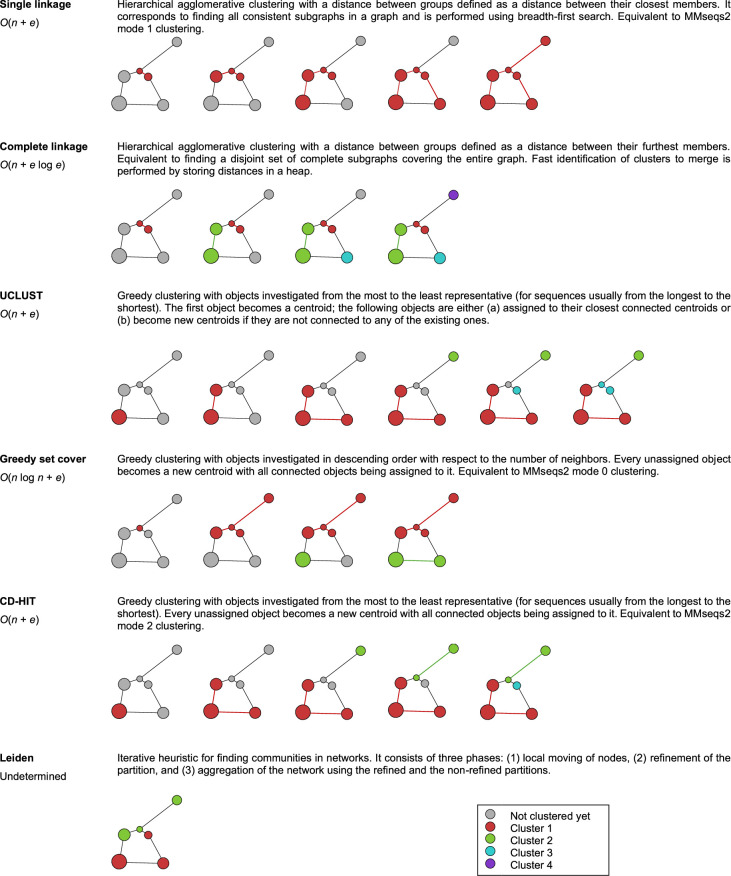
Clustering algorithms in Clusty. The dataset is represented as a graph: objects (genomes) are shown as vertices with sizes proportional to user-assigned weights (for example, sequence length) and edges indicate distances (for example, 1 – tANI). In the consecutive steps of each algorithm objects are assigned to clusters marked with distinct colors. The left panel shows each algorithm’s time complexity, with *n* as the number of vertices and *e* as the number of edges. Distance values for this example are available in the source data provided with the paper. Source data

**Extended Data Fig. 2:**
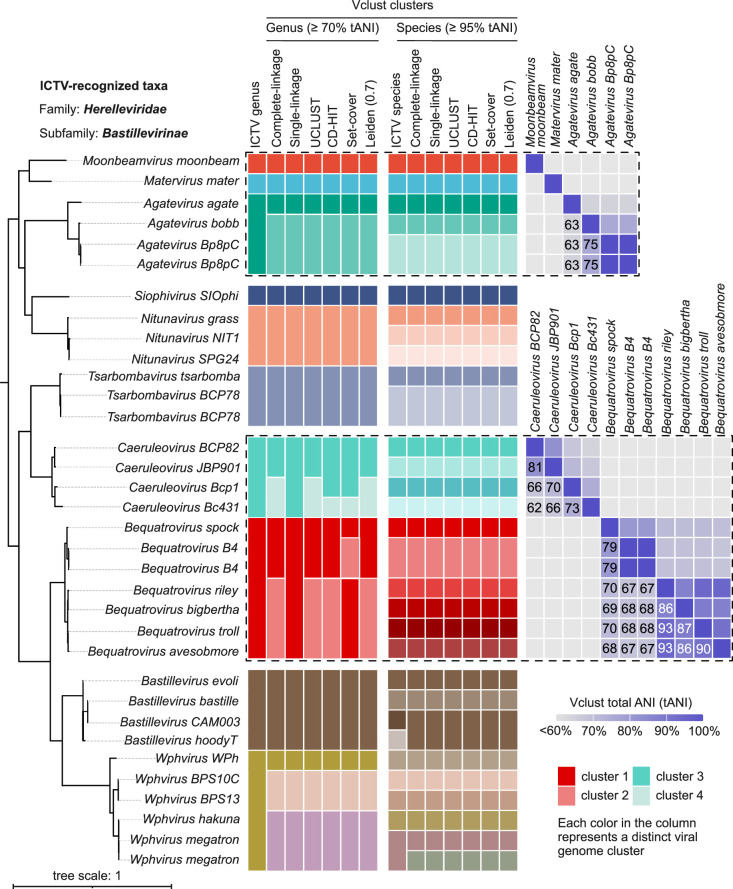
Vclust application to ICTV-recognized genera (threshold 70% tANI) and species (threshold 95% tANI). The subtree of *Bastillevirinae* branch from the maximum-likelihood tree based on a concatenated alignment of 10 marker proteins in *Herelleviridae* family. Leaves are labeled with species names and four species are represented by more than one genome (for example, multiple strains). Snippets of the total heatmap on the right highlight instances where Vclust clustering algorithms and/or ICTV disagree. Numbers in these boxes show tANI (only values higher than 60% - Vclust reliability threshold are shown). Despite recommended rank-demarcation criteria, the classification of viruses is based on multiple forms of evidence, ranging from calculating tANI between pairs to protein content and phylogenetic analyses. Therefore, inconsistencies between Vclust clustering and the official ICTV taxonomy may arise, especially if the tANI oscillates around the demarcation criterion, such as between *Bastillevirus hoodyT* and *Bastillevirus evoli*, where the tANI is 95.2%.

Clusty interprets input data as a graph, where vertices represent objects and edges represent connections. Extended Data Fig. 1 shows details of the clustering algorithms and their time complexities.

### Vclust implementation

Vclust is a Python tool integrating Kmer-db 2, LZ-ANI and Clusty for streamlined computation of intergenomic sequence similarities and clustering of viral genomes. Vclust provides three commands: ‘prefilter’, ‘align’ and ‘cluster’ (Fig. 1a). ‘prefilter’ and ‘align’ accept a single FASTA file containing viral genomic sequences or a directory of FASTA files (one genome per file), with support for gzipped inputs and outputs.

The ‘prefilter’ command uses Kmer-db 2 to screen out dissimilar genome pairs before alignment, reducing the number of genome pairs to only those with sufficient *k*-mer-based sequence similarity (that is, minimum number of common *k*-mers and/or the minimum sequence identity. Sequence identity in Kmer-db 2 is calculated similarly to ANI in Mash (1 − Mash distance) but uses the overlap coefficient^15^ instead of the Jaccard index. The overlap coefficient measures the intersection size of two *k*-mer sets (representing two genomic sequences) relative to the smaller set size, rather than the union of both sets. As a result, sequence identity values in the prefiltering step are generally higher than ANI from the alignment step. This allows users to set the minimum sequence identity in prefiltering close to the final ANI threshold without risking the exclusion of relevant genome pairs; for example, if targeting an ANI threshold of 95% or higher, the minimum sequence identity can be set to approximately 0.95 (Supplementary Fig. 4).

The ‘align’ command uses LZ-ANI to perform pairwise sequence alignments and compute ANI and AF measures between genome pairs identified by the pre-alignment filter. If the filter is not provided, Vclust aligns all possible genome pairs. The output includes two TSV files that are used for clustering: one containing ANI measures for genome pairs and the other listing genome identifiers sorted by decreasing sequence length. Optionally, Vclust can output detailed alignment results in a TSV format similar to BLASTn/MegaBLAST, with coordinates, strand orientation, matched/mismatched nucleotides and sequence identity for each local alignment.

The ‘cluster’ command uses Clusty for genome clustering, allowing users to specify a similarity measure (for example, tANI, ANI) and its threshold for clustering genomes, with optional additional filtering thresholds for other similarity measures, including AF. Output includes a TSV file listing genome identifiers and numerical cluster identifiers (including identifiers for singleton genomes). Alternatively, Vclust can output representative genomes instead of numerical cluster identifiers, which is particularly useful for dereplication tasks.

### Optimizing performance for highly redundant genome datasets

Vclust is designed for dereplication and clustering of viral sequences across a range of identity values. Computational performance may decline with datasets of highly redundant genome sequences (for example, tens of thousands of sequences from the same species; Supplementary Fig. 5). In all-versus-all pairwise genome comparisons in the ‘prefilter’ step, the high frequency of similar sequences expands the similarity matrix, increasing memory consumption and the number of pairs to align, which in turn raises computational demands for alignment and clustering. Vclust has three additional techniques to optimize performance and mitigate excessive resource consumption. First, it partitions a dataset into smaller, equally sized batches of genome sequences using the built-in multi-fasta-split C++ tool. This option considerably reduces memory requirements of the ‘prefilter’ step without altering results, although it may slightly increase runtime (Extended Data Fig. 4a). Second, Vclust can limit the number of *k*-mers analyzed from each genome sequence, reducing memory usage and runtime with minimal impact on sensitivity (Extended Data Fig. 4b). Third, similarly to MMseqs2 and BLAST-based methods, Vclust’s ‘prefilter’ can restrict the number of sequences reported per query genome by selecting those with the highest sequence identity, reducing the overall number of genome pairs passing initial similarity assessment.

### Benchmarking

#### Running time

All runtimes were benchmarked on a workstation equipped with an AMD Epyc 9554 CPU (64 cores clocked at 3.1 GHz) and 1,152 GiB (approximately 1,237 GB) RAM. Unless otherwise specified, all tools were run using 64 threads. The exact commands are shown in Supplementary Tables 8, 9 and 12.

#### Evaluating tANI accuracy

The tANI accuracy of Vclust v1.2.8, FastANI (v1.33)^5^, skani (v0.2.1)^6^ and VIRIDIC (v1.1)^4^ was assessed using two reference sets. In both reference datasets, VIRIDIC was run with default parameters (--word_size 7, --reward 2, --penalty 3, --gapopen 5, --gapextend 2) for highly sensitive BLASTn alignments. Similarly, skani was run in its most accurate mode optimized for small sequences (--slow, --s 0, --m 200). FastANI and Vclust were run with default parameters. The first reference dataset comprised 22,606 tANI values ranging from 70% to 100%, as determined by VIRIDIC across 4,244 complete genomes of bacteriophages affiliated with the ICTV using ICTV’s Virus Metadata Resource (VMR v38.3). Since FastANI and skani do not directly report tANI, their values were calculated from ANI, AF and genome lengths: tANI = (ANI_1_ × AF_1_ × LEN_1_ + ANI_2_ × AF_2_ × LEN_2_)/(LEN_1_ + LEN_2_). The second reference set contained expected (true) tANI values in the 70–100% range, derived from 10,000 pairs of bacteriophage genomes subjected to simulated mutations, including different levels of substitution, insertion, deletion, duplication, inversion and translocation events. Specifically, we randomly selected 100 genomes from the bacteriophage dataset and generated 100 copies of each genome. For each genome copy, we introduced mutations using Mutation-Simulator (v3.0.2)^16^ by randomly selecting a combination of mutation events and their corresponding frequencies (Supplementary Table 1). The expected (true) tANI value between each copy and reference genome was determined based on the variant call format produced by Mutation-Simulator, describing the exact locations of introduced mutations and the number of altered nucleotides.

#### Evaluating ANI and AF accuracy

The ANI and AF values predicted by Vclust, FastANI, skani, MegBLAST v2.13.0+ and MMseqs2 v2fad714b525f1975b62c2d2b5aff28274ad57466 (ref. ^9^) were compared to reference ANI and AF values determined by BLASTn (v2.13.0+)^8^. Since running BLASTn on the entire IMG/VR v4.1 database was not feasible, we subsampled 94,225 viral contigs and performed an all-to-all BLASTn search to identify 4,361,743 contig pairs meeting the MIUViG thresholds (ANI ≥ 95% and AF ≥ 85%). MegaBLAST, MMseqs and BLASTn outputs were used by the anicalc script from CheckV (v1.0.3)^3^ to compute ANI and AF values. Pearson correlation and MAE between the predicted and expected ANI and AF values were calculated based on the 4,361,743 contig pairs meeting MIUViG thresholds (ANI ≥ 95% and AF ≥ 85%) determined by BLASTn. Given the high level of sequence identity of the reference contig pairs, if a tool did not return a result for a given contig pair, the ANI and AF values were set to zero for that pair.

#### Evaluating clusterings

The agreement between clustering results from different tools and the reference clustering was assessed using the adjusted Rand index (ARI). ARI assesses clustering similarity by comparing the number of correct clustering overlaps and disagreements^17^ against those expected by chance. An ARI of 0 indicates random assignment, while a score of 1 indicates a perfect match. We used the scikit-learn (v1.3.2)^18^ implementation of the ARI.

### Reporting summary

Further information on research design is available in the Nature Portfolio Reporting Summary linked to this article.

## Online content

Any methods, additional references, Nature Portfolio reporting summaries, source data, extended data, supplementary information, acknowledgements, peer review information; details of author contributions and competing interests; and statements of data and code availability are available at 10.1038/s41592-025-02701-7.

## Supplementary information

Supplementary Information Supplementary Figs. 1–5

Reporting Summary

Peer Review File

Supplementary Tables 1–13 Supplementary Table 1. The expected (true) tANI values in the 70–100% range, derived from 10,000 pairs of bacteriophage genomes subjected to simulated mutations, including different levels of substitution, insertion, deletion, duplication, inversion and translocation events. Mutation-type frequencies and corresponding altered nucleotide counts are detailed in the table. Supplementary Table 2. Comparison of the tANI values predicted by Vclust and VIRIDIC in the reference to true ANI values in the ≥95% range. True tANI values were determined among 1,188 genome pairs with simulated mutations, including substitutions, insertions, deletions, inversions and translocations (Methods). Vclust shows superior accuracy in determining tANI, with a maximum difference (error) from the true tANI of 0.6%, whereas VIRIDIC's maximum error in tANI values reaches up to 4.7%. Supplementary Table 3. List of bacteriophage genome pairs showing tANI ≥ 95% as predicted by both Vclust and VIRIDIC, yet classified into different species by the ICTV. For each such pair, the history of all involved taxa was reviewed using the ICTV Taxonomy Browser and taxonomic proposals were checked for adopted demarcation criteria and presented. List of bacteriophage genome pairs showing tANI ≥ 95% as predicted by both Vclust and VIRIDIC, yet classified into different species by the ICTV. For each such pair, the history of all involved taxa was reviewed using the ICTV Taxonomy Browser (https://ictv.global/taxonomy/taxondetails/) and taxonomic proposals were checked for adopted demarcation criteria and presented evidence of genome-genome similarity. Supplementary Table 4. List of bacteriophage sequence pairs showing tANI < 95% as predicted by both Vclust and VIRIDIC, yet classified into same species by the ICTV. The identity of such records was reviewed by manual inspection of the GenBank records and associated literature. As our pipeline investigated complete genomes only, all sequences were interpreted as separate genomes. This fragmented genome was flagged, as the sequences belong to the same species, but show almost no sequence similarity. Supplementary Table 5. Comparison of species-level agreement between Vclust and other tools with the ICTV taxonomy (Virus Metadata Resource v38.3) across six different clustering algorithms. Clustering for the evaluated tools was conducted using Clusty based on a tANI threshold of ≥95%. Agreement between the tool clusterings and ICTV species-level clusters was assessed using the ARI, where an ARI of 0 represents no agreement beyond random chance, and an ARI of 1 indicates identical clustering results. Supplementary Table 6. List of 614 ICTV bacteriophage genera containing more than one species or genome. For each genus, the mean and maximum tANI values were calculated using VIRIDIC, the recommended and most widely used tool for genus delineation in ICTV taxonomic proposals. Notably, in nearly 10% of genera (*n* = 57), no genome pair exceeds the genus threshold of 70% tANI, and an additional 8% of genera (*n* = 43) have a mean tANI below 70%. Supplementary Table 7. List of 39 ICTV genera pairs with tANI values exceeding the genus threshold of 70%. The table includes the 609 pairs of genomes from different genera where tANI > 70%, encompassing 60 genera. Only the genome pairs (ID1 and ID2) with the highest tANI values are presented. Supplementary Table 8. Wall time and peak memory usage for all-to-all comparisons on the 4,244 bacteriophage genomes in Fig. 2b,c with 32 threads. The time and memory measurements of Vclust and VIRIDIC also include the clustering step, while FastANI and skani perform only ANI calculations. The table also contains the method commands and parameters used for benchmarking. Supplementary Table 9. Comparison of ANI and AF values predicted by the tools in reference to the expected ANI and AF values obtained from BLASTn. The ANI and AF values were calculated among 94,225 viral metagenomic contigs subsampled from IMG/VR v4.1. Only the contig pairs that satisfied the MIUViG’s threshold of ANI ≥ 95% and AF ≥ 85% were considered in the comparison. As shown in the third column, BLASTn returned 4,361,743 contig pairs satisfying the MIUViG’s threshold. The columns include the Pearson correlation coefficient (Pearson *r*) between the predicted and reference ANI/AF values, the MAE representing the average absolute difference between predicted and reference ANI/AF values, and the maximum error, which indicates the largest single absolute difference between a predicted ANI/AF value and its corresponding reference. Supplementary Table 10. Comparison of ANI and AF values predicted by the tools in reference to the expected ANI and AF values obtained from BLASTn. This table shows results of Supplementary Table 9, stratified by different contig sizes: <5 kb (*n* contigs = 19,280), 5–10 kb (*n* = 29,853), 10–20 kb (*n* = 17,238), 20–50 kb (*n* = 22,100), 50–100 kb (*n* = 4,747), >100 kb (*n* = 1,007). Only the contig pairs that satisfied the MIUViG's threshold of ANI ≥ 95% and AF ≥ 85% in BLASTn were considered in the comparison. Supplementary Table 11. Clustering 15,677,623 virus contig sequences from IMG/VR v4.1 into vOTUs based on the MIUViG thresholds (ANI ≥ 95% and AF ≥ 85%). As a reference, ANI and AF values between all-versus-all contigs were calculated from MegaBLAST (--max_target_seqs 20000 and --evalue 1e-03) and clustered with six algorithms using Clusty. The clustering outputs were evaluated against the MegaBLAST-based reference clustering using ARI (10th column). Additionally, comparisons were made with the original IMG/VR vOTU assignments (11th column). Notably, vOTU clusters based on ANI values from Vclust and MegaBLAST showed high ARI values across different clustering algorithms. Vclust with the ‘--kmers-fraction’ option set to 0.2, using 20% of *k*-mers during prefiltering, has minimal impact on the number of clusters compared to the default setting, which uses all *k*-mers. Discrepancies between the original IMG/VR vOTUs and Vclust/MegaBLAST clusters are due to differences in the implementation of the Leiden algorithm between the studies. vOTU clusters generated by each clustering algorithm were compared based on the distribution of intra-cluster and inter-cluster ANI values, as presented in Supplementary Fig. 1. Supplementary Table 12. Wall time and peak memory usage for calculating ANI and AF among 15,677,623 IMG/VR contigs, with 64 threads. Vclust performed *k*-mer-based estimation of sequence identity for 122,893,931,465,064 contig pairs (in the prefilter step), and aligned 811,099,152 contig pairs (in the ANI calculation step). BLASTn measurements were estimated from a random sample of 1,000 contigs used as a query to search a database of 15,677,623 contigs. ANI and AF values from MegaBLAST and BLASTn outputs were calculated using the anicalc tool from CheckV. Vclust with the ‘--kmers-fraction’ option set to 0.2, using 20% of *k*-mers during prefiltering, reduces runtime and RAM usage threefold compared to the default setting, which uses all *k*-mers. Supplementary Table 13. Selection of default LZ-ANI parameter values based on Bayesian optimization. The table summarizes the results of Bayesian optimization performed using scikit-optimize v0.9.0 on a dataset of 10,000 pairs of complete genomes, each with simulated mutations and known true tANI values of ≥70%. Each row represents one of the 100 evaluation runs, with different sets of LZ-ANI parameter combinations. The columns include the Pearson correlation coefficient (Pearson *r*) between the predicted and reference tANI values, the MAE representing the average absolute difference between predicted and reference tANI values, and the maximum error, which indicates the largest single absolute difference between a predicted tANI value and its corresponding reference. The evaluation run with the lowest MAE is highlighted in bold and is set as the default parameter configuration in Vclust.

## Source data

**Extended Data Fig. 3:**
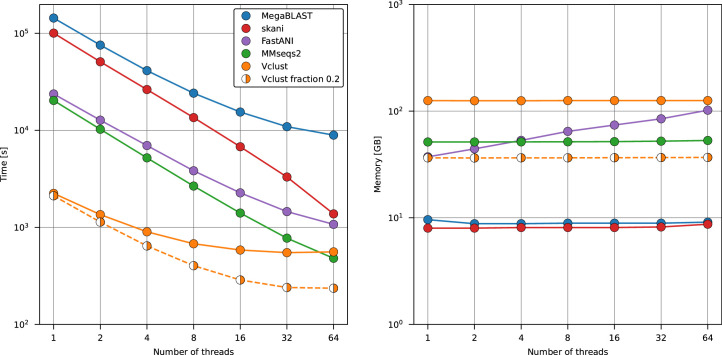
Wall time and peak memory usage of Vclust and other tools as a function of an increasing number of CPU threads on a dataset of 1 million metagenomic viral contigs. The dataset was sampled from IMG/VR v4.1, and the execution commands for the tools are detailed in Supplementary Table 12. Vclust was tested with all *k*-mers (default) and 0.2 fraction of *k*-mers retained at the *prefilter* step. The left panel shows execution times. The runtimes of all tools decrease linearly with up to eight threads, but beyond this, FastANI, MegaBLAST, and Vclust showed reduced speed-up while skani and MMseqs2 maintained linear scaling. Vclust with 0.2 *k*-mers fraction was the fastest algorithm independently of the number of threads. It was followed by the default Vclust setting with the exception of 64 threads, where slow-down caused by the task fragmentation overhead at the *prefilter* step made it inferior by a small margin to less accurate MMseqs2. In the right panel, memory usage was constant across thread counts for all algorithms except FastANI, which showed a linear increase in memory consumption. Source data

**Extended Data Fig. 4:**
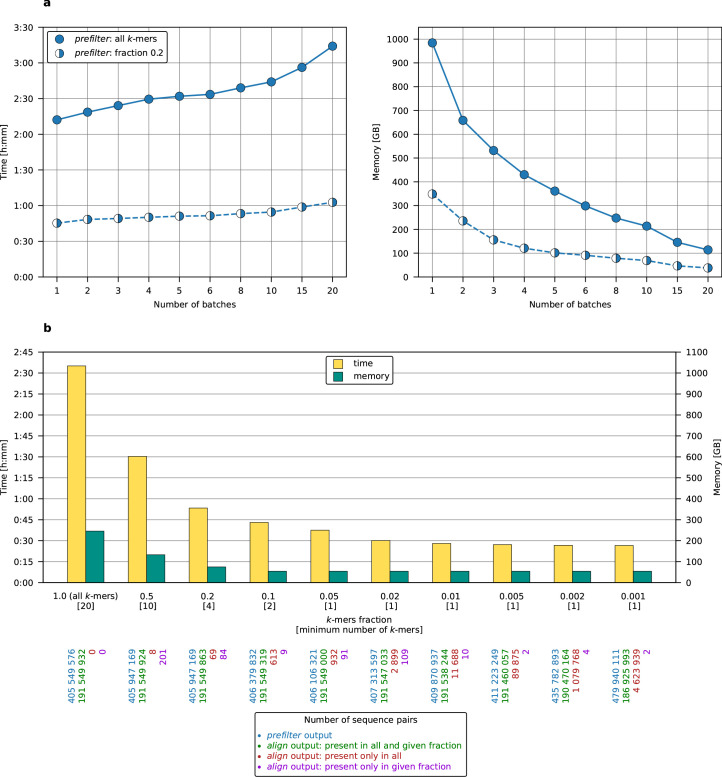
Performance of Vclust’s prefiltering step using the IMG/VR v4.1 dataset of 15,677,623 viral contigs. **a**, Wall time and peak memory usage for prefiltering genomes in a varying number of equally-sized sequence batches. The prefilter was configured to consider all *k*-mers (default) or 0.2 fraction of *k*-mers. Increasing the number of batches slightly increased runtime, significantly reducing memory usage for both *k*-mer fractions. **b**, Wall time and peak memory usage for different combinations of *k*-mers fractions (1 to 0.001) and minimum shared *k*-mers (20 to 1) during prefiltering. Below each combination, the outputs of *prefilter* and *align* steps are compared to the default settings (*k*-mers fraction 1 and minimum shared *k*-mers 20). The numbers represent: contig pairs passing the *prefilter* step (≥95% sequence identity; blue), pairs passing the *align* step (ANI ≥ 95%, AF ≥ 85%) common to the pairs obtained using all *k*-mers (green), contig pairs missed compared to using all *k*-mers (red), and additional contig pairs detected at the specified *k*-mers fraction but missing compared to using all *k*-mers (magenta). For instance, a 0.2 *k*-mers fraction reduces time and memory usage threefold, with a minimal impact on sensitivity and specificity. Source data

**Extended Data Fig. 5:**
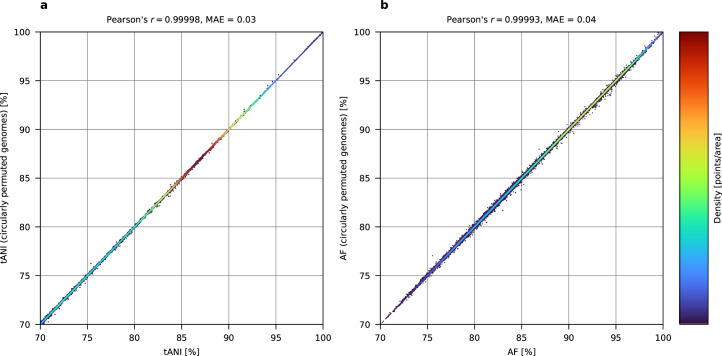
Evaluation of Vclust’s accuracy on circularly permuted genomes. Comparison of **a**, total average nucleotide identity (tANI) and **b**, aligned fraction (AF) values on a reference dataset of 4,244 complete bacteriophage genomes and the same dataset where two regions in each genome were swapped at a random breakpoint position. Both plots cover 40,618 genome pairs with tANI ≥ 70%. Source data

Source Data Statistical source data for Fig. 2b–f and Extended Data Figs. 1 and 3–5.

## Figures and Tables

**Extended Data Table 1:**
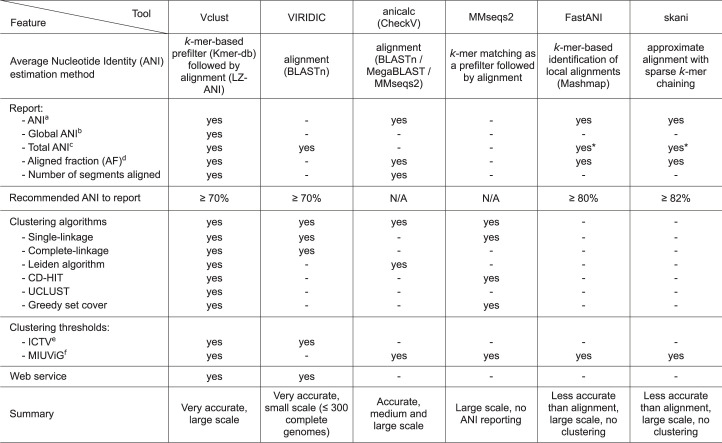
Comparison of Vclust features with other tools for sequence comparison and clustering of complete or metagenome-assembled virus genomes

^a^Number of identical bases across local alignments between two genomes divided by the total length of these alignments

^b^Number of identical bases across local alignments between two genomes divided by the length of the query/target genome

^c^Number of identical bases in local alignments between query-target and target-query genomes by the sum length of both genomes. This is equivalent to intergenomic similarity in VIRIDIC.

^d^Fraction of query/target genome aligned to one another

^e^International Committee on Taxonomy of Viruses (ICTV) recommends assigning viruses to the same species or genus based on total ANI of 95% and 70%, respectively.

^f^Minimum Information about an Uncultivated Virus Genome (MIUViG) standards recommend assigning viruses to virus operational taxonomic units based on 95% ANI over 85% alignment fraction (relative to the shorter sequence).

^*^Although total ANI is not directly reported, it can be approximated from ANI, alignment fraction, and genome lengths.

**Extended Data Table 2:**
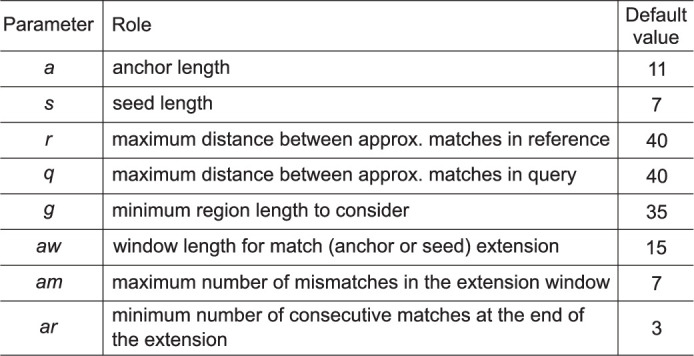
Parameters of LZ-ANI sequence aligner. All length values are given in nucleotides

## Data Availability

The datasets generated in this study have been deposited in in Figshare (10.6084/m9.figshare.28294805)^19^ and include complete RefSeq and GenBank genomes of 4,244 bacteriophages classified by ICTV, RefSeq and GenBank genome sequences of 10,000 bacteriophages with simulated mutations and corresponding expected total ANI values, and 94,225 metagenomic viral contigs sampled from IMG/VR v4.1 with expected BLASTn-based ANI and AF values. Supporting data generated in this study are provided in the Supplementary Information. Other databases used in the study include IMG/VR v.4.1 (https://genome.jgi.doe.gov/portal/IMG_VR/) and Virus Metadata Resource v38.3 from ICTV (https://ictv.global/vmr/). Source data are provided with this paper.

## Code availability

Vclust is available as a stand-alone tool at https://github.com/refresh-bio/vclust/ and 10.5281/zenodo.14756166 (ref. ^20^) and as a web service at https://www.vclust.org/. Kmer-db 2, LZ-ANI and Clusty are available in the GitHub repositories at https://github.com/refresh-bio/kmer-db/, https://github.com/refresh-bio/LZ-ANI/ and https://github.com/refresh-bio/clusty/, respectively.
