# Supplementary material for: Ultrafast and accurate sequence alignment and clustering of viral genomes
Source: Nat Methods. 2025 May 15;22(6):1191–4. doi: 10.1038/s41592-025-02701-7 (PMC12168504; doi:10.1038/s41592-025-02701-7)
Supplement: Supplementary file 1 — Supplementary Figs. 1–5 [file 41592_2025_2701_MOESM1_ESM.pdf]

nature methods

Supplementary information https://doi.org/10.1038/s41592-025-02701-7

Ultrafast and accurate sequence alignment and clustering of viral genomes

In the format provided by the authors and unedited

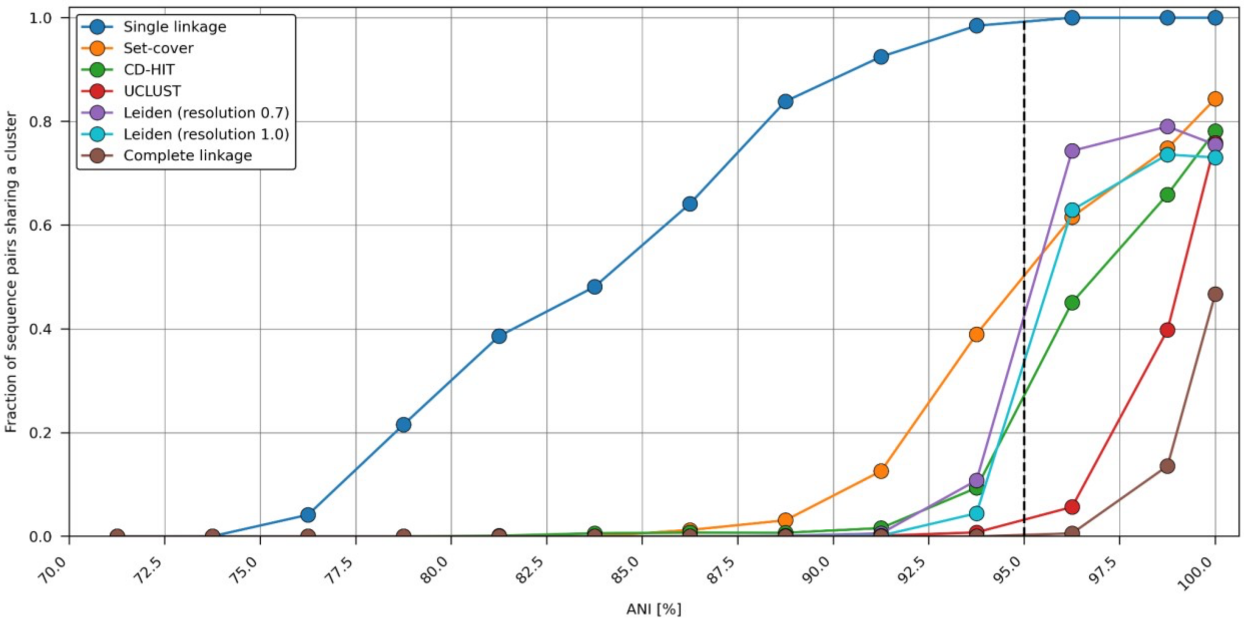

Supplementary Figure 1. Comparison of clustering algorithms for grouping metagenomic contigs into virus operational taxonomic units (vOTUs) based on the MIUViG-recommended thresholds of 95% average nucleotide identity (ANI) over 85% alignment fraction (AF). The line chart shows the proportion of contig pairs that are assigned to the same clusters (Y axis) across different ANI ranges (X axis). For example, nearly 50% of contig pairs within the ANI range from 82.5% to 85% are found in single-linkage clusters (blue), which were formed using the MIUViG thresholds (ANI ≥ 95% and AF ≥ 85%). Although single-linkage clusters contain all contig pairs with ANI values between 95% and 100%, they also include the highest proportion of contig pairs with ANI values below 95%. In contrast, complete-linkage clusters do not include any contig pairs with ANI below 95%, but they also contain the fewest pairs in the 95%-100% ANI range, indicating that many genome pairs with ANI ≥ 95% are either placed in separate clusters or exist as singletons (i.e., clusters with only one genome). The Leiden algorithm, tested with resolution parameters of 0.7 and 1.0 (brown and pink lines, respectively), appears most suitable for clustering vOTUs. It minimizes the inclusion of contig pairs with ANI values below the 95%, while including a significant number of pairs that meet or exceed this threshold. This makes the Leiden algorithm well-suited for maintaining the integrity of vOTUs while adhering to the MIUViG guidelines, and also supports the choice of this algorithm in the latest release of the IMG/VR v4.1 database. The analysis was performed on a reference dataset of 94,225 viral metagenomic contigs from the IMG/VR v4.1 database, as used in Figure 2d. The ANI and AF values were calculated using Vclust v1.1.0, and only contig pairs with AF ≥ 85% were included in the analysis.

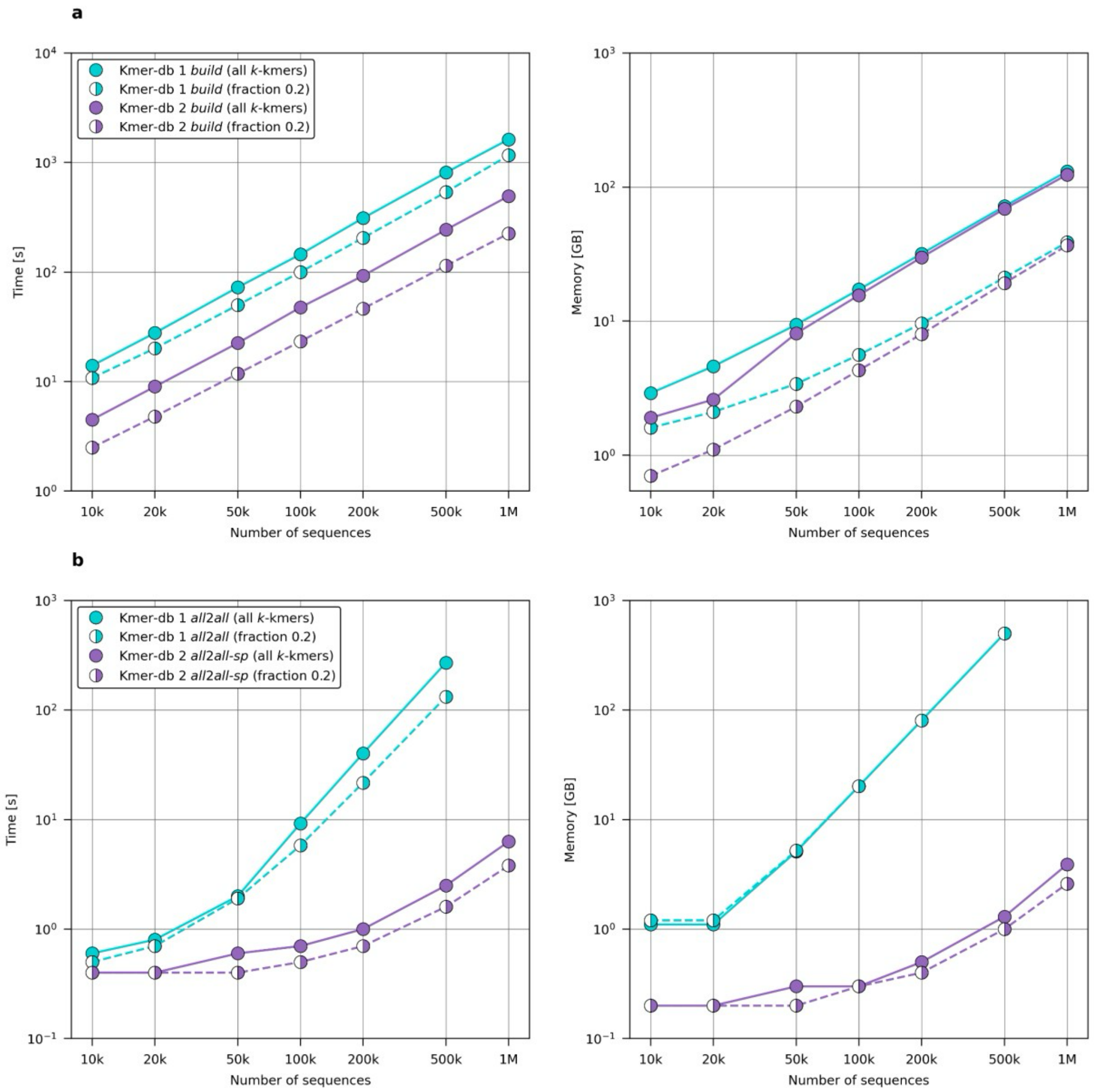

Supplementary Figure 2. Comparison of wall time and peak memory usage between Kmer-db 1 and Kmer-db 2 on subsampled metagenomic contig datasets from the IMG/VR v4.1 database, ranging from 10,000 to 1 million sequences. The algorithms were tested in two settings: using all *k*-mers (default) and a 0.2 fraction of *k*-mers. **a,** Construction of *k*-mer databases (*build* mode). With all *k*-mers, Kmer-db 2 was approximately 3x faster than Kmer-db 1 across all dataset sizes, with slightly reduced RAM usage. Using 0.2 fraction of *k*-mers reduced *build* times by ~30% (Kmerdb 1) and ~50% (Kmer-db 2) and noticeably decreased memory requirements (over 3-fold reduction for the largest dataset). **b,** Calculation of common *k*-mers for all genome pairs. The *all2all-sp* mode introduced in Kmer-db 2, optimized for sparse data, was over 100x faster than Kmer-db 1 a*ll2all* for datasets of 500,000 contig sequences. Using 0.2 fraction of k-mers reduced execution times of both algorithms by ~40% with the same (Kmer-db 1) or slightly lower (Kmerdb 2) memory footprint.

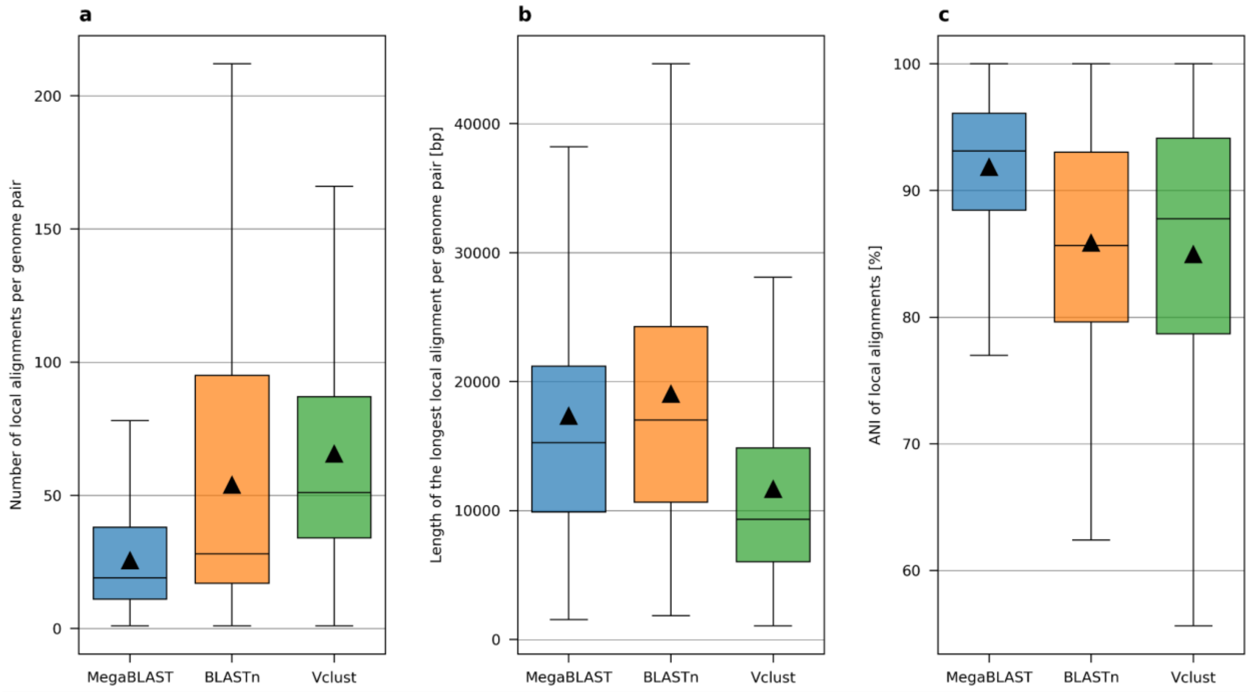

Supplementary Figure 3. Comparison of local alignments generated by Vclust, MegaBLAST, and BLASTn on

4,244 complete bacteriophage genomes, covering genome pairs with total ANI (tANI) ≥ 70%. Due to the asymmetry of average nucleotide identity (ANI), each genome pair is analyzed in both query-reference orientations (Fig. 1d), doubling the number of pairs (n = 45,214) compared to Fig. 2b. Vclust's local alignments, termed *regions*, are analogous to BLAST high-scoring pairs (HSPs) but ensure non-overlapping positions within the query sequences. At the nucleotide level, Vclust has a high level of agreement with BLASTn, with a 96% Jaccard similarity index (i.e., the proportion of nucleotides that are commonly aligned by both Vclust and BLASTn, relative to the total number of nucleotides aligned by either tool). **a,** Distribution of the number of local alignments per genome pair. On average, Vclust generates more alignments (mean = 65) than BLASTn (mean = 53) and MegaBLAST (mean = 25). **b,** Distribution of the length of the longest local alignment per genome pair. Vclust produces shorter alignments (mean = 11,644 bp) compared to BLASTn (mean = 19,008 bp) and MegaBLAST (mean = 17,300 bp). **c,** Distribution of ANI for local alignments. Vclust produces local alignments with comparable ANI to BLASTn (mean = 85%-86%). The boxplots show the median (central line), mean (triangle symbol), upper and lower quartiles (represented by the boxes), and the highest (upper whisker) and lowest (lower whisker) values within a 1.5 interquartile range.

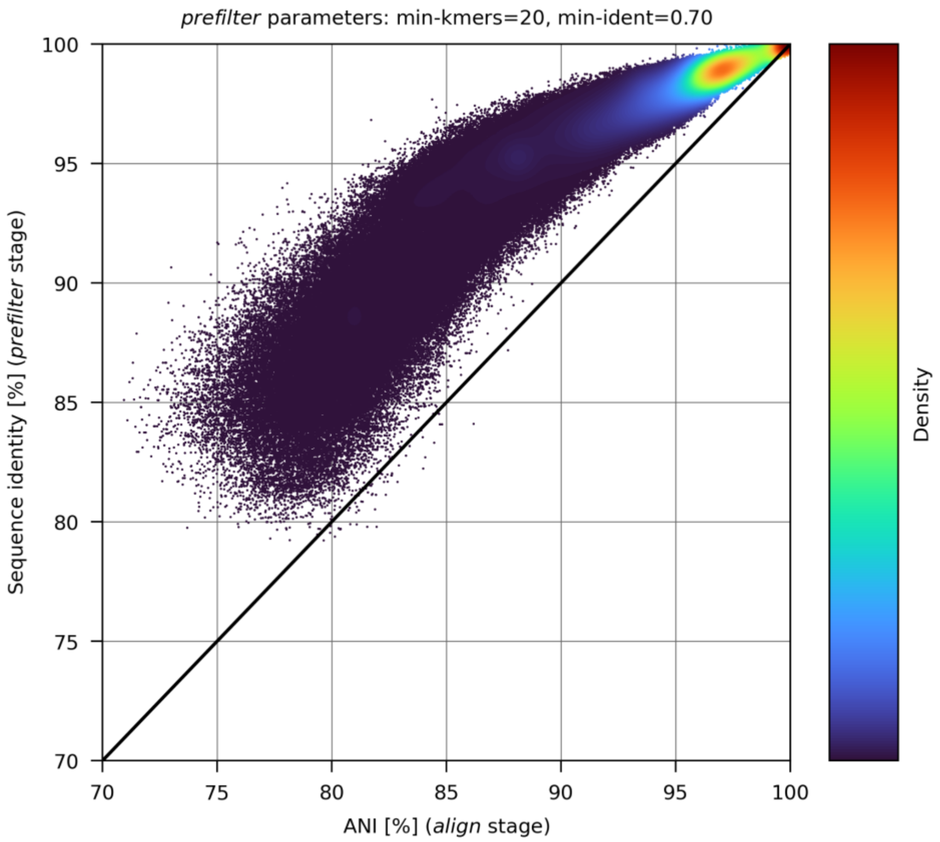

Supplementary Figure 4. Relationship between sequence identity from the Vclust's prefilter step and average nucleotide identity (ANI) from the alignment step. Sequence identity is generally higher than alignment-based ANI, allowing the minimum sequence identity in the prefiltering step to be set to closely approximate the final alignment-based ANI threshold. The analysis was performed on

5,025,568 contig pairs with ANI ≥ 70% from a dataset of 94,225 metagenomic viral contigs sampled from IMG/VR, as referenced in Figure 2d.

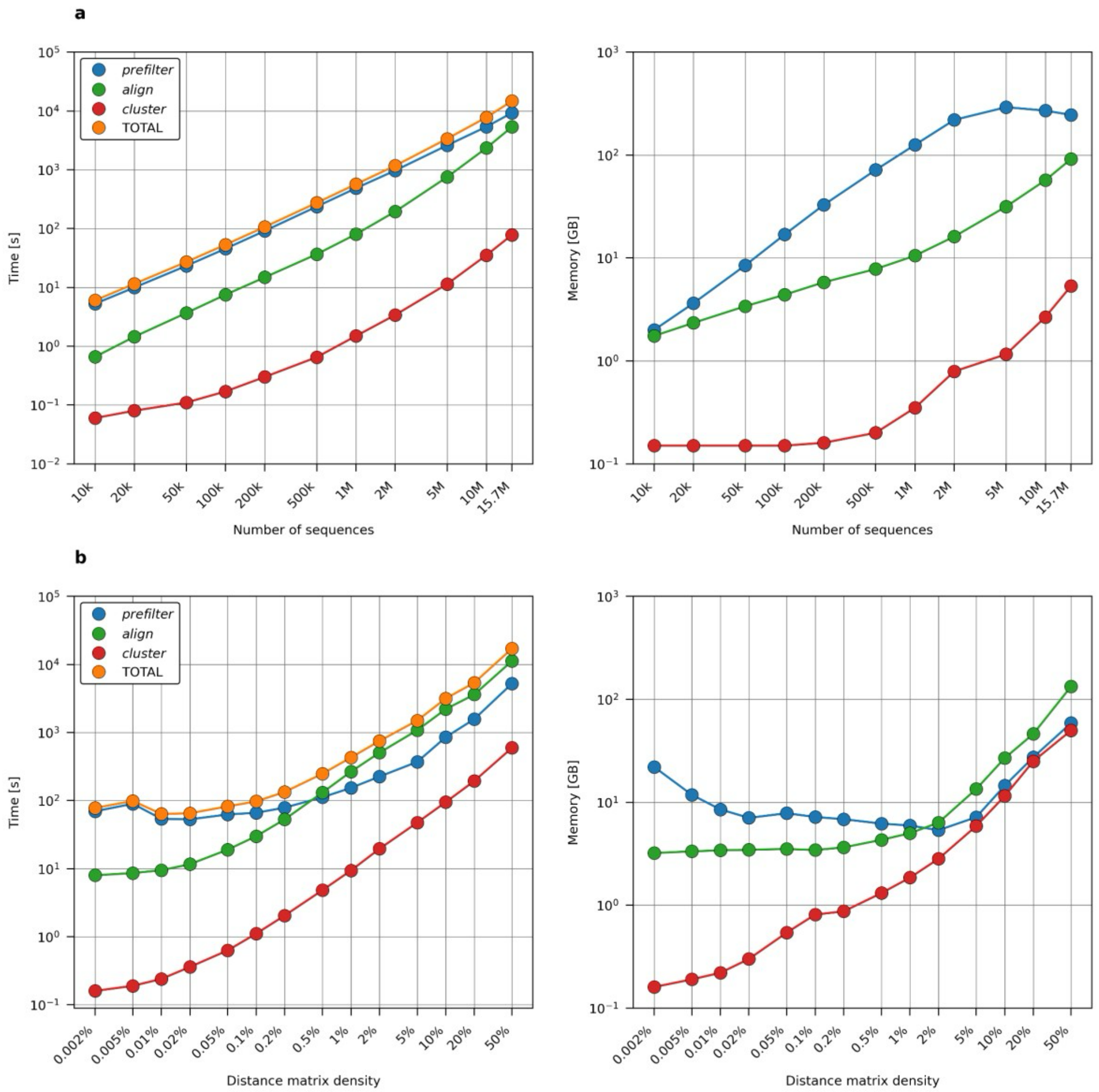

Supplementary Figure 5. Scalability of Vclust with increasing number of genome sequences and sequence relatedness. a, Wall time and peak memory usage for Vclust steps (*prefilter*, *align*, and *cluster*) across 11 datasets of increasing size, ranging from 10,000 to 15,677,623 metagenomic contigs subsampled from IMG/VR v4.1. Both runtime and RAM usage increased slightly faster-thanlinearly as the number of sequences increased. The memory consumption of the *prefilter* step plateaued at 2 million sequences, as Vclust handles larger datasets in batches. **b,** Wall time and peak memory usage for Vclust steps evaluated on 14 datasets (100,000 contigs each), with varying levels of genome relatedness (represented by increasing matrix density). For example, a 5% density dataset indicates that 5% of all pairs among 100,000 contigs exceed a 95% average nucleotide identity (ANI) threshold. This dataset was generated by randomly selecting 20 contigs from IMG/VR v4.1, each duplicated 5,000 times with small, simulated mutations to maintain the 95% ANI threshold. The runtime increased linearly from a matrix density of 0.5%. Beyond 2% density, the RAM usage for the *align* step became greater than that of the *prefilter*.
