# Supplementary material for: Ultrafast and accurate sequence alignment and clustering of viral genomes
Source: Nat Methods. 2025 May 15;22(6):1191–4. doi: 10.1038/s41592-025-02701-7 (PMC12168504; doi:10.1038/s41592-025-02701-7)
Supplement: Supplementary file 2 — Reporting Summary [file 41592_2025_2701_MOESM2_ESM.pdf]

nature portfolio

Corresponding author(s): Sebastian Deorowicz
Bas E. Dutilh

Last updated by author(s): Jan 28, 2025

Reporting Summary

Nature Portfolio wishes to improve the reproducibility of the work that we publish. This form provides structure for consistency and transparency in reporting. For further information on Nature Portfolio policies, see our Editorial Policies and the Editorial Policy Checklist.

Statistics

For all statistical analyses, confirm that the following items are present in the figure legend, table legend, main text, or Methods section.

n/a	Confirmed	
☐	☒	The exact sample size (*n*) for each experimental group/condition, given as a discrete number and unit of measurement
☐	☒	A statement on whether measurements were taken from distinct samples or whether the same sample was measured repeatedly
☒	☐	The statistical test(s) used AND whether they are one- or two-sided<br>*Only common tests should be described solely by name; describe more complex techniques in the Methods section.*
☒	☐	A description of all covariates tested
☒	☐	A description of any assumptions or corrections, such as tests of normality and adjustment for multiple comparisons
☐	☒	A full description of the statistical parameters including central tendency (e.g. means) or other basic estimates (e.g. regression coefficient) AND variation (e.g. standard deviation) or associated estimates of uncertainty (e.g. confidence intervals)
☒	☐	For null hypothesis testing, the test statistic (e.g. *F*, *t*, *r*) with confidence intervals, effect sizes, degrees of freedom and *P* value noted<br>*Give P values as exact values whenever suitable.*
☒	☐	For Bayesian analysis, information on the choice of priors and Markov chain Monte Carlo settings
☒	☐	For hierarchical and complex designs, identification of the appropriate level for tests and full reporting of outcomes
☐	☒	Estimates of effect sizes (e.g. Cohen's *d*, Pearson's *r*), indicating how they were calculated

Our web collection on statistics for biologists contains articles on many of the points above.

Software and code

Policy information about availability of computer code

Data collection	No software was used.
Data analysis	Our tool Vclust 1.2.8 (described in the manuscript and available at https://github.com/refresh-bio/vclust), integrating Kmer-db 2.2.3 (https://github.com/refresh-bio/kmer-db), LZ-ANI 1.2.2 (https://github.com/refresh-bio/lz-ani), and Clusty 1.1.3 https://github.com/refresh-bio/clusty, was used for data analysis. Benchmarking was performed against Viridic 1.1, FastANI 1.33, skani 0.2.1, MMseqs2 v2fad7, MegaBLAST 2.13+, and BLASTn 2.13+, and the anicalc Python script from CheckV 1.0.3. Simulated datasets of bacteriophage genomes were prepared using Mutation-Simulator 3.0.2. The adjusted Rand Index was calculated with scikit-learn v1.3.2.

For manuscripts utilizing custom algorithms or software that are central to the research but not yet described in published literature, software must be made available to editors and reviewers. We strongly encourage code deposition in a community repository (e.g. GitHub). See the Nature Portfolio guidelines for submitting code & software for further information.

Data

Policy information about availability of data

All manuscripts must include a data availability statement. This statement should provide the following information, where applicable:

- Accession codes, unique identifiers, or web links for publicly available datasets
- A description of any restrictions on data availability
- For clinical datasets or third party data, please ensure that the statement adheres to our policy

The datasets generated in this study have been deposited in in Figshare (https://doi.org/10.6084/m9.figshare.28294805) and include complete RefSeq and GenBank genomes of 4,244 bacteriophages classified by ICTV, RefSeq and GenBank genome sequences of 10,000 bacteriophages with simulated mutations and corresponding expected total ANI values, and 94,225 metagenomic viral contigs sampled from IMG/VR v4.1 with expected BLASTn-based ANI and AF values. Supporting data generated in this study are provided in the Supplementary Information, Source Data and Supplementary Data files. Other databases used in the study include IMG/VR v.4.1 (https://genome.jgi.doe.gov/portal/IMG_VR/) and Virus Metadata Resource v38.3 from ICTV (https://ictv.global/vmr). Source data are provided with this paper.

Human research participants

Policy information about studies involving human research participants and Sex and Gender in Research.

Reporting on sex and gender	N/A (not human research)
Population characteristics	N/A (not human research)
Recruitment	N/A (not human research)
Ethics oversight	N/A (not human research)

Note that full information on the approval of the study protocol must also be provided in the manuscript.

Field-specific reporting

Please select the one below that is the best fit for your research. If you are not sure, read the appropriate sections before making your selection.

☒ Life sciences ☐ Behavioural & social sciences ☐ Ecological, evolutionary & environmental sciences

For a reference copy of the document with all sections, see nature.com/documents/nr-reporting-summary-flat.pdf

Life sciences study design

All studies must disclose on these points even when the disclosure is negative.

Sample size	Sample-size calculations were not performed. In most experiments, the entire datasets were used, with the following exceptions:<br>* For estimating BLASTN runtime on the IMG/VR dataset, 1,000 contigs were randomly selected.<br>* For the simulated dataset, 100 bacteriophage genomes were randomly chosen from the ICTV dataset, and 100 variants were generated for each using Mutation-Simulator.<br>* For estimating ANI/AF accuracy, 94,225 viral contigs were randomly selected from the IMG/VR dataset.
Data exclusions	No data were excluded from the analyses. Complete datasets served as benchmarks. Additionally, for one reference dataset containing bacteriophage genomes from ICTV, we manually created a subset of high-quality species groupings by excluding genome pairs without sufficient evidence in ICTV's proposals supporting their classification as a single species. The excluded genome pairs are listed in Tables S4 and S5.
Replication	Testing was conducted on various machine configurations, including Linux and Windows x64-based desktops, Linux x64-based servers, MacOS x64-based desktops, and MacOS ARM-based desktops. The results were consistent across all configurations.
Randomization	We propose a new method for calculating ANI and clustering. All methods were applied to all datasets, except for BLASTN on the entire IMGVR dataset, which was infeasible due to an estimated runtime of over four years. Therefore, for estimating BLASTN runtime, we randomly selected 1,000 contigs and extrapolated the results.
Blinding	Not applicable, as this paper focuses on method development and benchmarking.

Reporting for specific materials, systems and methods

We require information from authors about some types of materials, experimental systems and methods used in many studies. Here, indicate whether each material, system or method listed is relevant to your study. If you are not sure if a list item applies to your research, read the appropriate section before selecting a response.

Materials & experimental systems

n/a	Involved in the study
☒	☐ Antibodies
☒	☐ Eukaryotic cell lines
☒	☐ Palaeontology and archaeology
☒	☐ Animals and other organisms
☒	☐ Clinical data
☒	☐ Dual use research of concern

Methods

n/a	Involved in the study
☒	☐ ChIP-seq
☒	☐ Flow cytometry
☒	☐ MRI-based neuroimaging
